# Epidemiological, clinical, and forensic approach to a series of defendants examined in criminal psychiatric expertise

**DOI:** 10.1192/j.eurpsy.2024.1209

**Published:** 2024-08-27

**Authors:** M. Kacem, W. Bouali, M. Abdelaziz, S. Brahim, L. Zarrouk

**Affiliations:** Psychiatric department, Taher Sfar Hospital of Mahdia, Mahdia, Tunisia

## Abstract

**Introduction:**

The transgression of the law can be a deliberate act by a lucid adult, but it can also be the result of a deficiency in judgment and discernment due to lack of age or insanity. Psychiatric expertises are more and more solicited in the penal field with the objective of identifying the causal link between mental illness and the criminal act.

**Objectives:**

The objective of this work was to give an overview of the subjects expertised, the offences and the pathologies encountered through the report of a psychiatric expertise activity in criminal law.

**Methods:**

It is a retrospective descriptive study carried out on the criminal psychiatric expertises made for a forensic act in the psychiatric department of Mahdia during the period from January 1, 2003 to March 30, 2022.

**Results:**

In total, we collected 101 defendants. The average age of our study population was 35±12.07 years. The majority of our study sample was male (98%), from an urban area (50%). 46.6% had primary education and only 11.2% had higher education. The defendants were single in 57.8% of the cases, and almost half (45.7%) had no occupation. Two-thirds of the accused (61.2%) had experienced emotional deprivation at a young age. The problematic use of psychoactive substances was found in 10.3%, as well as alcohol consumption in 46.6%. Moreover, 39.7% of the patients had a personal psychiatric history and 19% had been incarcerated at least once. The forensic acts were mostly against people (62.9%) dominated by physical aggression (33.6%) followed by homicide or its attempt in 19% of the cases. The majority of patients were not related to their victims (62.1%). The nosographic diagnosis found was a personality disorder in 32.75% of cases, followed by schizophrenic disorders in 22.4% of cases.

**Conclusions:**

Psychiatric expertise is a useful, complex and noble clinical act. Determining the predictive factors of a possible acting out allows to specify the objectives of interventions aiming at limiting the acts of violence, hospitalizations and incarcerations of patients suffering from mental disorders.

**Disclosure of Interest:**

None Declared

